# *Sphingobium yanoikuyae* Bacteremia, Japan

**DOI:** 10.3201/eid3005.231514

**Published:** 2024-05

**Authors:** Yayoi Miyamatsu, Ryutaro Tanizaki, Satoko Yamada

**Affiliations:** Ise Municipal General Hospital, Ise, Japan

**Keywords:** *Sphingobium yanoikuyae*, bacteremia, antibacterial agents, antimicrobial drugs, ceftriaxone, ceftazidime, bacteria, Japan

## Abstract

We report a case of *Sphingobium yanoikuyae* bacteremia in an 89-year-old patient in Japan. No standard antimicrobial regimen has been established for *S. yanoikuyae* infections. However, ceftriaxone and ceftazidime treatments were effective in this case. Increased antimicrobial susceptibility data are needed to establish appropriate treatments for *S. yanoikuyae*.

The genus *Sphingomonas* was divided into 4 clusters, and *Sphingomonas yanoikuyae* was renamed *Sphingobium yanoikuyae* (*1*). *S. yanoikuyae* is a gram-negative, nonsporulating, strictly aerobic rod-shaped bacterium (*2*) widely distributed in natural environments, especially in water and soil, and is rarely a human pathogen (*3*). Although 1 case of *S. yanoikuyae* infection has been reported in the central nervous system (CNS) of a child (*4*), infections have not been reported in adults. We report a case of *S. yanoikuyae* bacteremia in an older man.

An 89-year-old man from Japan sought care at an emergency department because of fever and chills lasting 1 hour. He had been taking prednisolone (5 mg/day) for 6 years for interstitial pneumonia. He was alert, and his vital signs were as follows: body temperature, 38.6°C; heart rate, 71 beats/min; blood pressure, 112/64 mmHg; respiratory rate, 28 breaths/min; and blood oxygen saturation, 100% while breathing room air. Laboratory findings revealed elevated leukocyte count (16,100 cells/μL; reference range 3,300–8,600 cells/μL) and C-reactive protein level (4.16 mg/dL; reference range 0–0.14 mg/dL) but were otherwise unremarkable. Chest computed tomography revealed honeycombing and multiple reticular shadows in both lungs, unchanged from 5 months earlier. We suspected sepsis and administered intravenous ceftriaxone (2 g/24 h) after obtaining 2 sets of blood samples for culture. On day 2, the patient’s fever subsided. On day 5, a blood culture sample yielded positive results after incubation in an aerobic BACTEC Plus Aerobic/F Culture Vial in a BACTEC FX system (Becton Dickinson, https://www.bd.com). Gram staining revealed small gram-negative rods (Figure, panel A) that we were unable to identify by using mass spectrometry (MALDI Biotyper; Bruker Daltonics, https://www.bruker.com). We subsequently cultured the positive blood culture fluid on Trypticase Soy Agar with 5% Sheep Blood (Becton Dickinson) at 35°C in an aerobic environment and identified *S. yanoikuyae* by using mass spectrometry of bacteria isolated on day 6 (Figure, panels B, C). Genetic analysis of a 1,402 nt 16S rRNA sequence revealed 99.5% homology with *S. yanoikuyae* (Appendix). We performed antimicrobial susceptibility testing by using the dilution method and a Neg MIC NF1J panel (Beckman Coulter, https://www.beckmancoulter.com) in accordance with Clinical and Laboratory Standards Institute (CLSI) criteria for other non-Enterobacterales bacteria (Table) (*5*). We determined the ceftriaxone MIC by using the Neg MIC EN 2J panel for Enterobacterales bacteria and Pos MIC 1J panel for gram-positive cocci (both Beckman Coulter). Although *S. yanoikuyae* was susceptible to ceftriaxone, we preferred to use antimicrobial drugs that were effective against glucose nonfermenting bacteria, which is the fermentation pattern exhibited by *Sphingomonas* spp. On day 6, we switched the antimicrobial to ceftazidime (1 g/8 h). We did not detect *S. yanoikuyae* in blood cultures at follow-up on days 6 and 11, indicating treatments were effective, and the patient’s condition remained stable. However, severe aspiration pneumonia developed on day 16, and he died of respiratory failure on day 17.

**Figure:**
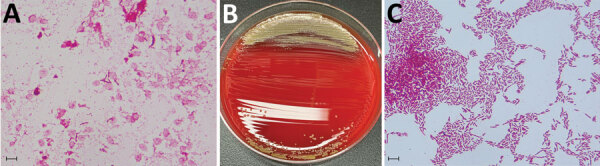
Identification of *Sphingobium yanoikuyae* bacteremia in 89-year-old man, Japan. A) Gram stain of the organisms growing in a blood sample incubated in a BACTEC Plus Aerobic/F Culture Vial (Becton Dickinson, https://www.bd.com). Scale bar is 10 μm. B) Colonies of *S. yanoikuyae* cultured on Trypticase Soy Agar with 5% Sheep Blood (Becton Dickinson). C) Gram stain of *S. yanoikuyae* bacteria from a colony obtained by subculturing positive blood culture fluid on Trypticase Soy Agar with 5% Sheep Blood at 35°C in an aerobic environment. Scale bar is 10 μm.

**Table:** Drug susceptibility pattern for *Sphingobium yanoikuyae* isolated from an 89-year-old man’s blood sample in study of *S. yanoikuyae* bacteremia, Japan*

Antimicrobial drug	MIC†, µg/mL	Breakpoint MIC‡, µg/mL
Piperacillin/tazobactam	<4/4	16/4
Ceftriaxone	4	8
Ceftazidime	2	8
Cefepime	<1	8
Aztreonam	>16	8
Imipenem	1	4
Meropenem	4	4
Gentamicin	<1	4
Tobramycin	<1	4
Amikacin	<4	16
Minocycline	<1	4
Ciprofloxacin	<0.25	1
Levofloxacin	<0.5	2
Trimethoprim/sulfamethoxazole	<1/19	2/38

*Drug susceptibility data according to Clinical and Laboratory Standards Institute criteria (*5*). MIC values for antimicrobial drugs, except ceftriaxone, were determined by using a Neg MIC NF1J panel (Beckman Coulter, https://beckmancoulter.com). The MIC value of ceftriaxone was determined by using Neg MIC EN 2J Enterobacterales and Pos MIC 1J gram-positive cocci panels (both Beckman Coulter). †MIC for the isolate from 89-year-old case-patient. ‡Breakpoints for other non-Enterobacterales susceptible strains.

Within the genus *Sphingomonas*, *S. paucimobilis* is the most frequently reported cause of human infection (*6*), predominantly causing bacteremia, septicemia, peritonitis, lung infections, pneumonia, or urinary tract infections; 24 of 52 (46%) cases in published literature were of nosocomial origin (*7*). Thus, *Sphingomonas* spp. might be a chief cause of nosocomial infection in addition to other glucose nonfermenting bacteria. The *S. yanoikuyae* infection reported previously in a child was a nosocomial infection after head surgery (*4*). Although this case in an older man was not a nosocomial infection, he had been taking prednisolone for 6 years, which might have increased his infection risk.

No antimicrobial regimen has been established for treating *S. yanoikuyae* infections. The child who had a CNS infection received 28 days of intravenous meropenem and 5 days of intrathecal amikacin (*4*). A novel bacteria strain, CC4533, isolated from a contaminated Tris-acetate-phosphate agar plate used to grow *Chlamydomonas reinhardtii*, showed 99.55% DNA sequence identity to *S. yanoikuyae*; drug susceptibility testing indicated CC4533 was resistant to polymyxin B, penicillin, and chloramphenicol and sensitive to neomycin (*8*). We treated our patient with intravenous ceftriaxone and then ceftazidime. Cefepime, a 4th-generation cephalosporin, can penetrate the cerebral spinal fluid and has an additional quaternary ammonium group enabling penetration through the outer membrane of gram-negative bacteria, increasing effectiveness against β-lactamase–producing gram-negative bacilli (*9*). We selected ceftazidime, a 3rd-generation cephalosporin, because our clinical findings did not suggest a CNS infection, and *S. yanoikuyae* did not produce β-lactamase.

No breakpoints have been established for *Sphingobium* sp. bacteria; thus, we evaluated antimicrobial susceptibility according to CLSI criteria for other non-Enterobacterales bacteria (*5*). According to the dilution method, MIC values for ceftriaxone were >2 by using the Enterobacterales panel and <4 by using the gram-positive cocci panel. The ceftriaxone MIC for the isolate from this patient was 4, which is below the CLSI breakpoint of 8 for other non-Enterobacterales bacteria (*5*), indicating that the isolate was susceptible to ceftriaxone.

In conclusion, no standard antimicrobial treatment regimen has been established for *S. yanoikuyae*. Ceftriaxone and ceftazidime were effective treatments for *S. yanoikuyae* infection in this patient. Increased antimicrobial susceptibility data are needed to establish appropriate treatments for *S. yanoikuyae*. 

Appendix Additional information for *Sphingobium* *yanoikuyae* bacteremia, Japan.
