# Supplementary material for: Sphingobium yanoikuyae Bacteremia, Japan
Source: Emerg Infect Dis. 2024 May;30(5):1060–2. doi: 10.3201/eid3005.231514 (PMC11060447; doi:10.3201/eid3005.231514)
Supplement: Appendix — Additional information for Sphingobium yanoikuyae bacteremia, Japan. [file 23-1514-Techapp-s1.pdf]

Article DOI: https://doi.org/10.3201/eid3005.231514

EID cannot ensure accessibility for supplementary materials supplied by authors. Readers who have difficulty accessing supplementary content should contact the authors for assistance.

Sphingobium yanoikuyae Bacteremia, Japan

Appendix

16S rRNA Sample Sequences

Sample_518F Signal G:1282 A:1220 C:1612 T:1227
Lane: 45 Base Spacing: 14.643044 964 bases in 11647 scans
GGGGAAAAAGGTTTTCGGATTACTGGGCGTAAGCGCACGTAGGCGGCTATTCAAGT
CAGAGGTGAAAGCCCGGGGCTCAACCCCGGAACTGCCTTTGAAACTAGATAGCTTG
AATCCAGGAGAGGTGAGTGGAATTCCGAGTGTAGAGGTGAAATTCGTAGATATTCG
GAAGAACACCAGTGGCGAAGGCGGCTCACTGGACTGGTATTGACGCTGAGGTGCGA
AAGCGTGGGGAGCAAACAGGATTAGATACCCTGGTAGTCCACGCCGTAAACGATGA
TAACTAGCTGTCAGGGCACATGGTGTTTTGGTGGCGCAGCTAACGCATTAAGTTATC
CGCCTGGGGAGTACGGTCGCAAGATTAAAACTCAAAGGAATTGACGGGGGCCTGCA
CAAGCGGTGGAGCATGTGGTTTAATTCGAAGCAACGCGCAGAACCTTACCAACGTTT
GACATCCCTATCGCGGATCGTGGAGACACTTTCCTTCAGTTCGGCTGGATAGGTGAC
AGGTGCTGCATGGCTGTCGTCAGCTCGTGTCGTGAGATGTTGGGTTAAGTCCCGCAA
CGAGCGCAACCCTCGCCTTTAGTTGCCAGCATTTAGTTGGGTACTCTAAAGGAACCG
CCGGTGATAAGCCGGAGGAAGGTGGGGATGACGTCAAGTCCTCATGGCCCTTACGC
GTTGGGCTACACACGTGCTACAATGGCGACTACAGTGGGCAGCCACCTCGCGAGAG
GGAGCTAATCTCCAAAAGTCGTCTCAGTTCGGATCGTTCTCTGCAACTCGAGAGCGT
GAAGGCGGAATCGCTAGTAATCGCGGATCAGCATGCCGCGGTGGAATACCGTTCCC
AGGCCTTGTACACACCGCCCGTCACACCATGGGAGTTGGATTCACTCGAAGGCGTTG
AGCTAACCGTAAGGAGGCAGGCGACCACAGTGGGTTTAGCGACTGGGGTGAGCTAG
AGGGA
Sample_800R Signal G:1287 A:1340 C:2903 T:1931
Lane: 43 Base spacing: 14.576325 706 bases in 8462 scans
2GGCCCTTTTTCGCCCTCAGCGTCATACCAGTCCAGTGAGCCGCCTTCGCCACTGGTG
TTCTTCCGAATATCTACGAATTTCACCTCTACACTCGGAATTCCACTCACCTCTCCTG
GATTCAAGCTATCTAGTTTCAAAGGCAGTTCCGGGGTTGAGCCCCGGGCTTTCACCT
CTGACTTGAATAGCCGCCTACGTGCGCTTTACGCCCAGTAATTCCGAACAACGCTAG
CTCCCTCCGTATTACCGCGGCTGCTGGCACGGAGTTAGCCGGAGCTTATTCTCCCGG
TACTGTCATTATCATCCCGGGTAAAAGAGCTTTACAACCCTAAGGCCTTCATCACTC
ACGCGGCATTGCTGGATCAGGCTTTCGCCCATTGTCCAATATTCCCTACTGCTGCCTC
CCGTAGGAGTCTGGGCCGTGTCTCAGTCCCAGTGTGGCTGATCATCCTCTCAGACCA
GCTAAGGATCGTCGCCTTGGTGAGCCTTTACCTCACCAACTAGCTAATCCTACGCGG

GCTCATCCTTGGGCGATAAATCTTTGGACTTACGTCATCATCCGGTATTAGCTTCCGT
TTCCAGAAGTTATTCCGAACCCAAGGGCAGATTCCCACGCGTTACGCACCCGTGCGC
CACTATCTCCGAAGAGATCGTTCGACTTGCATGTATTAGGCATGCCGCCAGCGTTCG
TTCTGAGCAGTTCAAAAAA
